# Exploring the relationship between dietary quality, eating behavior, and mental health among young adults

**DOI:** 10.3389/fnut.2025.1598260

**Published:** 2025-11-03

**Authors:** Michaela Poslt Königová, Martina Sebalo Vňuková, Ivan Sebalo, Veronika Koleničová, Lucie Urbanová, Petra Řehořková, Martin Anders

**Affiliations:** Department of Psychiatry, First Faculty of Medicine, Charles University and General University Hospital, Prague, Czechia

**Keywords:** mental health, young adults, dietary quality, disordered eating, eating behaviors, cross-sectional study

## Abstract

**Background:**

Nutritional psychiatry has established that nutrient-dense diets rich in fruits, vegetables, whole grains, and lean proteins are associated with lower rates of depression and anxiety, while diets high in refined sugars and saturated fats predict greater psychological distress. Although emotion-regulation strategies are known to influence eating behavior, evidence on their role in shaping overall diet quality remains scarce, particularly in Central and Eastern European populations. Young adulthood (18–30 years) represents a critical developmental stage in which both mental health vulnerabilities and long-term dietary patterns consolidate, yet no prior study has examined how discrete cognitive emotion-regulation strategies relate to both global diet quality and specific eating behaviors in Czech young adults.

**Methods:**

In the Czech Republic, we conducted a cross-sectional survey of 1,027 young adults (507 men, 520 women; mean age = 24.6, SD = 3.3 years) recruited via quota-based convenience sampling matched to the 2021 census on age (18–30 years), sex, education, and region. Data were collected in three 60-min online sessions. Participants completed validated Czech measures of depression (BDI), anxiety (BAI), psychological distress (SCL-90), burnout (Shirom-Melamed Burnout Measure), and nine CERQ subscales—all demonstrating *α* ≥ 0.89 in prior validations and *α* ≥ 0.79 in our pilot (*N* = 50). Diet quality was assessed using a 30-item Czech FFQ (pilot α = 0.81), from which Diet Quality Index International (DQI-I) scores were computed. Eating behaviors were measured with the 31-item Czech Adult Eating Behavior Questionnaire (AEBQ; α_total = 0.79). We used stepwise multiple regression with all variance inflation factors < 2.0 to identify psychological variables associated with DQI-I and with “food-approach” versus “food-avoidance” behaviors, minimizing overfitting.

**Results:**

In exploratory stepwise regression models (all variance inflation factors < 2.0), the DQI-I model explained a small proportion of variance (Adj R^2^ = 0.024; f^2^ = 0.025). Within this model, higher rumination was positively associated with diet quality (B = 0.34, *p* < 0.001), while depressive symptoms were inversely associated with diet quality (B = −0.09, *p* = 0.001). The AEBQ model accounted for a modest but meaningful share of variance (Adj R^2^ = 0.155; f^2^ = 0.183). In this model, anxiety, catastrophizing, and “focus on the positive” were positively associated with food-approach behaviors (all *p* < 0.001), whereas positive reappraisal and acceptance negatively associated with dysregulated eating (*p* < 0.01). These associations should be regarded as tentative and hypothesis-generating, given the exploratory design and modest variance explained.

**Conclusion:**

This study is the first census-matched study of Czech young adults to examine emotion-regulation strategies in relation to both diet quality and eating behaviors. These findings reveal complex and partly counterintuitive associations—for example, rumination was linked to healthier diet quality, while some ostensibly adaptive strategies coincided with more dysregulated eating. These results should be interpreted as exploratory and hypothesis-generating, underscoring the potential relevance of cognitive-emotional mechanisms for future nutritional psychiatry interventions. Integrating approaches that address maladaptive strategies such as catastrophizing with dietary guidance may represent a promising direction for prevention and health-promotion efforts but requires confirmation in longitudinal and experimental studies.

## Introduction

Since Hibbeln’s (1) Lancet report found that populations consuming more fish exhibited up to 20% lower depression rates—likely via omega-3 fatty acids’ modulation of serotonergic and dopaminergic pathways—nutritional psychiatry has emerged. Observational cohorts (2, 3, 39) and meta-analyses (4, 43) consistently link nutrient-dense dietary patterns, i.e., high in fruits, vegetables, whole grains, lean proteins, and key micronutrients to reduced depression and anxiety, whereas diets rich in refined sugars and saturated fats exacerbate psychological distress (37). Mechanistic research further implicates micronutrients such as B vitamins (in homocysteine metabolism) and amino acids (in neurotransmitter synthesis) as biological mediators of these diet–mental health relationships (5, 6, 40, 41).

Young adulthood (ages 18–30) represents a critical developmental period characterized by transitions in education, employment, and independent living. During this time, dietary patterns often shift away from home-cooked meals toward more convenience and processed foods, with long-term consequences for both physical and psychological health. Central and Eastern European data suggest that young adults increasingly prefer processed foods and irregular eating patterns (7, 8), yet few studies have examined how these changes interact with mental health.

Beyond nutrient intake, emotion regulation may play a pivotal role in shaping dietary behaviors. Maladaptive strategies such as rumination and catastrophizing have been linked to stress-related overeating and impulsive food choices (9, 10), whereas adaptive approaches such as positive reappraisal and acceptance are thought to buffer against stress-induced dysregulated eating (11). However, existing evidence is inconsistent, and little is known about whether discrete emotion-regulation strategies predict overall dietary quality, specific eating-behavior patterns, or both.

To date, no large-scale, census-matched study has investigated these relationships in Czech young adults. This is a critical gap as this population navigates psychosocial transitions within a rapidly changing Central European food environment. Understanding the links between mental health, emotion regulation, and diet during this period may provide insights for targeted prevention and intervention strategies.

The present study therefore examined how depressive and anxiety symptoms, along with selected Cognitive Emotion Regulation Questionnaire (CERQ) subscales (rumination, catastrophizing, positive reappraisal, acceptance), relate to nutritional outcomes: (a) overall dietary quality assessed by the Diet Quality Index–International (DQI-I), and (b) eating behaviors assessed by the Adult Eating Behavior Questionnaire (AEBQ) “food-approach” versus “food-avoidance” subscales in a quota-based sample matched to the 2021 Czech census. We tested three hypotheses: (1) H1: maladaptive emotion-regulation strategies (rumination and catastrophizing) and higher depressive or anxiety symptoms would predict poorer diet quality; (2) H2: these same maladaptive factors would predict higher food-approach and lower food-avoidance behaviors on the Adult Eating Behavior Questionnaire (AEBQ); and (3) H3: adaptive strategies (positive reappraisal, acceptance) would attenuate the negative associations observed in H1 and H2, reflecting a protective role.

By integrating validated mental-health and emotion-regulation measures with culturally adapted dietary and eating-behavior assessments in a large, census-matched Czech cohort, this study seeks to advance understanding of the complex pathways connecting emotion regulation, diet, and mental health in Central and Eastern Europe.

## Methods

### Sample

Between March and June 2023, we surveyed 1,027 Czech young adults (507 men [49.4%], 520 women [50.6%]; mean age = 24.6 years, SD = 3.3). Participants were recruited through quota-based convenience sampling from STEM MARK’s national panel, stratified to match the 2021 Czech census on age (18–30 years), sex (male/female), education (primary, secondary, tertiary), and region (Bohemia, Moravia, Silesia) (12).

The inclusion criteria were age 18–30 years, residence in the Czech Republic, and fluency in Czech. The exclusion criteria were current or past psychiatric diagnoses, screened via a 5-item DSM-5 screener (13). Ethics approval (project C793401) was granted by the General Faculty Hospital in Prague before data collection, and informed consent was obtained from all participants.

Data collection was organized into three 60-min online waves between March and June 2023. Every participant (*N* = 1,027) completed all three waves; no additional invitation and attrition data were available from STEM MARK.

The measures included in each wave were as follows: Wave 1 (31 March 2023; Protocol 2,619, version 32): sociodemographic and socioeconomic status (age, sex, education, region, income), health history (medical conditions, medications) and sexual activity, Shirom-Melamed Burnout Measure, physical-activity questionnaire, Czech FFQ and AEBQ; Wave 2 (14 May 2023; Protocol 2,683, version 24): ACEs, perceived stress and social support, BDI, BAI, SCL-90, WHOQOL-BREF, aggression, CERQ subscales, climate-change worry item; and finally Wave 3 (26 June 2023; Protocol 2,734, version 16): healthy-lifestyle principles (smoking, substance use), spirituality, body image, sleep quality, and personality traits.

Although the broader project encompassed comprehensive lifestyle and mental-health assessments, this manuscript focuses exclusively on the Wave 1 dietary and eating-behavior measures and the Wave 2 mental-health and emotion-regulation measures.

### Measures

#### Eating behavior and diet

Eating behaviors were assessed using the Adult Eating Behavior Questionnaire (AEBQ) (14, 15, 34, 35, 36, 38), originally a 35-item self-report measure comprising eight subscales. For this study, we used a Czech adaptation in which four items with low item total correlations (< 0.30) or communalities (< 0.25) in a pilot sample (*N* = 50) were removed, resulting in a 31-item version. Items are rated on a 5-point Likert scale (1 = strongly disagree to 5 = strongly agree). Based on theoretical structure and our pilot factor analysis, we created two aggregated scales: Food Approach (Food Responsiveness, Enjoyment of Food, Emotional Overeating; pilot *α* = 0.82) and Food Avoidance (Satiety Responsiveness, Emotional Undereating, Food Fussiness; pilot α = 0.76).

Original AEBQ norms (15) report subscale alphas of 0.70–0.90 and mean scores approximately 3.0 (SD ≈ 0.8), providing benchmarks for comparison. For each participant, we calculated the mean score of the items comprising each aggregated scale, where higher values indicate a stronger tendency toward that pattern of eating behavior.

Dietary intake was measured using a semi-quantitative, 30-item Czech Food Frequency Questionnaire (FFQ), developed by a nutrition specialist at the General Faculty Hospital in Prague for use with young adults. Participants reported their frequency of consumption over the past month for major food groups (e.g., fruits, vegetables, whole grains and legumes, proteins, snacks, and sweets and/or beverages) on a scale ranging from “never” to “several times per day.” Portion sizes were estimated by participants. Although this FFQ has not undergone full validation in Czech populations, pilot resting (*N* = 50) demonstrated internal consistency (*α* = 0.81) and test–retest reliability (r = 0.78 over 2 weeks).

Diet quality was operationalized using the Diet Quality Index–International (DQI-I) (16) computed from FFQ responses. The DQI-I comprises four domains: (1) Variety (0–20): number of food groups and protein sources consumed, (2) Adequacy (0–40): intake of fruits, vegetables, grains, fiber, protein, calcium, and iron, (3) Moderation (0–30): limits on total fat, saturated fat, cholesterol, sodium, and empty-calorie foods, and (4) Balance (0–10): macronutrient ratio and fatty-acid composition. Total scores range from 0 (poor diet quality) to 100 (optimal diet quality). Domain-specific scores allow for targeted interpretation (e.g., low variety but high moderation).

#### Mental health instruments

To capture a broad spectrum of mental-health dimensions, participants completed the following Czech-validated self-report measures:

- Beck Depression Inventory (BDI-II) (17): A 21-item measure of depressive symptom severity over the past 2 weeks (*α* = 0.89) (18).
- Beck Anxiety Inventory: A 21-item scale assessing the intensity of anxiety symptoms during the past week (*α* = 0.92) (18).
- Symptom Checklist-90: A 90-item inventory of general psychological distress across nine symptom dimensions (α = 0.95) (19).
- Shirom-Melamed Burnout Measure: A 14-item scale evaluating physical fatigue, cognitive weariness, and emotional exhaustion (α = 0.89) (20, 42).
- World Health Organization Quality of Life: Four domains (physical health, psychological health, social relationships, environment), of which we used the physical and psychological subscales (αs = 0.82 and 0.85, respectively) (18).
- Cognitive Emotion Regulation Questionnaire (CERQ) (11): a 36-item measure of nine cognitive coping strategies (e.g., rumination, catastrophizing, and positive reappraisal; subscale αs = 0.72–0.84 in Czech pilot).

All instruments demonstrated strong internal consistency in prior Czech validations and in our pilot testing, ensuring reliable assessment of depressive and anxiety symptoms, general distress, burnout, quality of life, and cognitive emotion-regulation strategies.

### Hypothesis

Drawing on prior evidence linking emotion regulation, eating behavior, and diet quality to mental health, we tested three hypotheses:

H1: Psychological Predictors of Diet Quality: we hypothesized that greater use of maladaptive emotion-regulation strategies (rumination, catastrophizing) and higher symptoms of depression and anxiety would be associated with poorer diet quality, as measured by the Diet Quality Index–International (DQI-I).

H2: Psychological Predictors of Eating Behaviors: we hypothesized that the same maladaptive strategies and mental-health symptoms would predict distinct patterns of eating behavior—specifically, higher “food-approach” and lower “food-avoidance” scores on the Adult Eating Behavior Questionnaire (AEBQ).

H3 (Exploratory): Protective Role of Adaptive Coping: we explored whether adaptive emotion-regulation strategies (positive reappraisal, acceptance) would attenuate the associations in H1 and H2, such that individuals scoring higher on these coping styles would exhibit better diet quality and more regulated eating behaviors despite higher psychological symptoms.

Although mediation and bidirectionality were of conceptual interest, our cross-sectional design and analytic plan (stepwise regression) focus on identifying direct psychological predictors of DQI-I and AEBQ outcomes; formal mediation tests are beyond the scope of this study.

## Results

### Analysis

All analyses were conducted in Jamovi v2.3.2. Total item missingness ranged from 0.2 to 4.7% across variables and was handled via multiple imputation (m = 20) using predictive mean matching. Univariate outliers (any value > |3| SD from the mean) were excluded from regression analyses (*n* = 12 cases), yielding a final analytic sample of *N* = 1,015.

We first evaluated internal consistency (Cronbach’s *α*) for all measures and computed means, standard deviations, and zero-order Pearson’s correlations among key variables to inspect bivariate relationships. Variance inflation factors (VIFs) for all predictors were < 2.0, indicating low multicollinearity.

To test hypotheses, we conducted two sets of stepwise multiple regression analyses:

1. Predicting overall diet quality (DQI-I total score),
2. Predicting eating behaviors (AEBQ Food Approach and Food Avoidance scales).

Predictors included depressive and anxiety symptoms (BDI-II, BAI), burnout (SMBM), distress (SCL-90), selected WHOQOL subscales, and CERQ subscales (rumination, catastrophizing, positive reappraisal, acceptance, focus on the positive, positive refocusing, and social relationships).

Model selection was guided by Akaike’s information criterion (AIC) to balance fit and parsimony. For each final model, we report unstandardized coefficients (B), standardized coefficients (*β*), t-values, *p*-values, adjusted R^2^, and Cohen’s f^2^.

To test Hypothesis 3, we conducted sensitivity analyses including interaction terms between adaptive coping strategies (positive reappraisal, acceptance) and mental-health symptoms.

We report unstandardized coefficients (B), standardized coefficients (β), t-values, *p*-values, adjusted R^2^, and Cohen’s f^2^. Given the use of stepwise regression, the findings should be interpreted as exploratory and hypothesis-generating rather than confirmatory. All tests were two-tailed with *α* = 0.05.

### Descriptive statistics

The full sample comprised 1,027 Czech young adults, including 507 men (49.4%) and 520 women (50.6%), with ages ranging from 18 to 30 years (M = 24.6, SD = 3.3). In terms of educational attainment, one participant (0.1%) had completed only primary school; 155 (15.1%) had some secondary education without a final exam; 434 (42.3%) held a secondary school diploma; 125 (12.2%) had earned a bachelor’s degree; 139 (13.5%) held a master’s degree; and one participant (0.1%) reported having a postdoctoral qualification.

Anthropometric data revealed that men (*n* = 507) had a mean weight of 81.7 kg (SD = 13.2), a mean height of 179.5 cm (SD = 7.6), and a mean BMI of 25.4 (SD = 3.2). Women (*n* = 520) had a mean weight of 67.7 kg (SD = 11.5), a mean height of 165.9 cm (SD = 6.5), and a mean BMI of 24.6 (SD = 4.1).

Key study variables in the full sample included a mean Diet Quality Index–International score of 59.8 (SD = 12.4, range 0–100) and mean Adult Eating Behavior Questionnaire scores of 2.89 (SD = 0.38) for Food Approach and 3.12 (SD = 0.34) for Food Avoidance. Mental-health measures showed a mean BDI score of 12.4 (SD = 7.8) and a mean BAI score of 9.2 (SD = 6.5).

After excluding 12 univariate outliers (|z| > 3), an independent-samples *t*-test with Welch’s correction (*N* = 1,015) revealed that women (M = 2.98, SD = 0.34) scored significantly higher than men (M = 2.79, SD = 0.39) on the AEBQ Food Approach scale (t(996.6) = −8.15, *p* < 0.001, Cohen’s d = 0.51). No significant sex difference was found for the Food Avoidance scale (t(1,013) = 1.32, *p* = 0.19).

### Exploratory factor analysis (EFA) of the Czech AEBQ

An exploratory factor analysis (EFA) was conducted on the 31-item Czech Adult Eating Behavior Questionnaire (AEBQ) using maximum likelihood extraction with oblimin rotation. Seven factors emerged, cumulatively explaining 48.86% of the total variance (Table 1). Factor loadings were all ≥ 0.30 on their primary factor, and item uniqueness values ranged from 0.18 to 0.80 (Table 2).

**Table 1:** Factor variance summary.

Factor	SS loadings	% of variance	Cumulative %
1	2.60	8.66	8.66
2	2.49	8.30	16.95
3	2.23	7.42	24.37
4	2.14	7.15	31.52
5	1.82	6.05	37.57
6	1.72	5.73	43.30
7	1.67	5.55	48.86

Factors 1–7 cumulatively explain 48.86% of the variance.

**Table 2:** Factor loadings from exploratory factor analysis using maximum likelihood extraction and oblimin rotation.

Item	Factor 1	Factor 2	Factor 3	Factor 4	Factor 5	Factor 6	Factor 7	Uniqueness
i34_4	0.878							0.233
i34_3	0.852							0.235
i34_1	0.778							0.413
i34_33	0.304							0.805
i34_29		0.902						0.179
i34_14		0.795						0.358
i34_25		0.774						0.279
i34_26		0.477						0.552
i34_32			0.741					0.498
i34_28			0.620					0.591
i34_13			0.421					0.661
i34_22			0.419					0.622
i34_6			0.417					0.801
i34_17			0.396					0.568
i34_34			0.321					0.729
i34_9			0.301					0.787
i34_16				0.837				0.300
i34_8				0.815				0.362
i34_5				0.631				0.525
i34_18					0.769			0.410
i34_35					0.761			0.399
i34_15					0.638			0.511
i34_12						0.814		0.291
i34_7						0.738		0.416
i34_2						0.463		0.664
i34_24						0.416		0.728
i34_31							0.649	0.485
i34_23							0.545	0.612
i34_30							0.472	0.689
i34_11							0.383	0.640

“Maximum likelihood” extraction method was used in combination with a “oblimin” rotation.

Model fit indices indicated an excellent fit: the root mean square error of approximation was 0.047 (90% CI [0.043, 0.050]), below the conventional cutoff of 0.05, and the Tucker–Lewis Index was 0.91, exceeding the recommended threshold of 0.90. The negative Bayesian information criterion (BIC = −907) further supported this solution over alternative factor structures, despite a significant chi-square statistic (χ^2^(246) = 800, *p* < 0.001) reflecting sensitivity to sample size (Table 3).

**Table 3:** Model fit measures.

	RMSEA 90% CI				Model test		
RMSEA	Lower	Upper	TLI	BIC	χ^2^	df	*p*
0.0467	0.0432	0.0504	0.910	−907	800	246	< 0.001

RMSEA = root mean square error of approximation; TLI = Tucker–Lewis Index; BIC = Bayesian information criterion; χ^2^ = chi-square statistic; df = degrees of freedom. The 90% confidence interval for RMSEA was 0.0432 to 0.0504.

These results confirm that the adapted Czech AEBQ captures seven distinct dimensions of eating behavior with acceptable psychometric properties, validating its use for subsequent regression analyses.

### Confirmatory factor analysis (CFA) of the Czech AEBQ

The confirmatory factor analysis of the 31-item Czech AEBQ demonstrated excellent fit to the theoretical model (see Table 3). Specifically, the root mean square error of approximation (RMSEA) was 0.047, with a 90% confidence interval ranging from 0.043 to 0.050—below the conventional cutoff of 0.05. The Tucker–Lewis Index (TLI) was 0.91, exceeding the recommended threshold of 0.90. The Bayesian information criterion (BIC) was −907, indicating superior fit relative to competing models. Although the chi-square statistic was significant (χ^2^(246) = 800, *p* < 0.001), the ratio of χ^2^ to degrees of freedom (χ^2^/df = 3.25) remained within acceptable bounds. Together, these indices confirm that the adapted Czech AEBQ closely replicates the original factor structure.

### Pearson’s correlations among DQI, AEBQ, WHOQOL, CERQ, and mental health scales—the third hypothesis

Preliminary Pearson’s correlations (*N* = 1,030) examined associations among Diet Quality Index–International (DQI-I), overall eating behaviors (AEBQ total), mental-health indices (BDI, BAI, SCL-90, Shirom-Melamed), quality of life (WHOQOL physical, psychological, social, environmental), and emotion-regulation strategies (CERQ subscales).

First, diet quality showed small but significant associations with mental-health and wellbeing measures: Higher DQI-I correlated with lower depressive symptoms (BDI; r = −0.084, *p* = 0.007) and marginally with lower burnout (Shirom-Melamed; r = −0.038, *p* = 0.276, ns). DQI-I was unrelated to anxiety (BAI; r = −0.021, *p* = 0.497, ns) but positively associated with environmental quality of life (WHOQOL Environment; r = 0.111, *p* < 0.001) and with adaptive coping strategies—Positive Reappraisal (r = 0.130, *p* < 0.001) and Planning (r = 0.134, *p* < 0.001)—suggesting that individuals with healthier diets tend to report better environmental wellbeing and greater use of constructive emotion-regulation.

Second, eating-behavior dysregulation (AEBQ total) was moderately linked to psychological distress: AEBQ total correlated with depressive symptoms (BDI; r = 0.237, *p* < 0.001), anxiety (BAI; r = 0.263, *p* < 0.001), overall distress (SCL-90; r = 0.266, *p* < 0.001), and burnout (Shirom-Melamed; r = 0.243, *p* < 0.001). Higher AEBQ scores also related to lower physical (WHOQOL Physical; r = −0.080, *p* = 0.010) and psychological (WHOQOL Psychological; r = −0.117, *p* < 0.001) quality of life.

Third, quality of life domains showed strong inverse relationships with distress: WHOQOL Physical and Psychological correlated with BDI in the range r = −0.44 to −0.55 (all *p* < 0.001) and with SCL-90 (r = −0.44 to −0.48, *p* < 0.001), indicating that better perceived wellbeing accompanies fewer symptoms.

Finally, emotion-regulation strategies demonstrated the expected pattern: Maladaptive CERQ subscales (Rumination, Self-Blame, Catastrophizing) were positively associated with distress (e.g., Rumination–SCL-90: r = 0.363, *p* < 0.001; Self-Blame–BDI: r = 0.397, *p* < 0.001), whereas adaptive strategies showed protective links (positive reappraisal–BDI: r = −0.071, *p* = 0.022; acceptance–BDI: r = −0.130, *p* < 0.001).

These results support our hypotheses that healthier diets and adaptive coping correspond with lower distress and higher quality of life, while dysregulated eating and maladaptive coping align with greater psychological symptoms. Full correlation matrices are presented in Supplementary Table S1.

### Testing the first hypothesis

To identify which psychological variables best predict diet quality (DQI-I), we conducted a stepwise multiple regression using the Akaike information criterion for variable selection and ensuring all variance inflation factors remained < 2.0. The initial model included the following predictors: Beck Anxiety Inventory (BAI), Shirom-Melamed Burnout Measure (Shirom Total), SCL, selected subscales from the WHOQOL questionnaire, and six CERQ subscales (Focus on the Positive, Positive Reappraisal, Positive Refocusing, Acceptance, Catastrophizing, and Social Relationships). The primary goal was to determine how these variables relate to DQI and which of them provide the greatest contribution to explaining its variance.

In the exploratory stepwise model, the overall association with DQI was small but statistically significant (R = 0.163, Adj. R^2^ = 0.024, *F*(2,803) = 10.95, *p* < 0.001). These results indicate that collectively, the selected predictors accounted for a very small proportion of variance in DQI and should be interpreted as hypothesis-generating (see Table 4).

**Table 4:** Model summary for stepwise regression predicting DQI-I.

R	R square	Adjusted R square	Std. error of the estimate
0.163^b^	0.027	0.024	8.10

^b^ Indicates variables that were retained as statistically significant predictors in the final stepwise regression model.

The results of the analysis of variance (ANOVA) indicate that the model is statistically significant (*F*(2, 803) = 10.949, *p* < 0.001). This outcome suggests that, taken together, the predictors make a significant contribution to explaining the variability in the dependent variable, DQI (see Table 5).

**Table 5:** ANOVA for stepwise regression predicting DQI-I.

Variable	Sum of squares	df	Mean square	F	Sig.
Regression	1436.44	2	718.22	10.95	0.000^c^
Residual	52673.95	803	65.60		
Total	54110.39	805			

A regression analysis revealed that several variables significantly contributed to the variance in the dependent variable, DQI. The intercept was 49.960, representing the baseline DQI level when all other predictors are zero. Rumination was positively associated with DQI (B = 0.341, *β* = 0.153, t = 4.182, *p* < 0.001) in the exploratory model, indicating that higher levels of rumination are associated with higher DQI scores. In contrast, greater depressive symptoms (BDI) were inversely associated with DQI (B = −0.090, β = −0.120, t = −3.279, *p* = 0.001), suggesting that higher BDI scores correspond to lower DQI scores (see Table 6).

**Table 6:** Coefficients.

Variable	Unstandardized coefficients B	Std. error	Standardized coefficients beta	t	Sig.
(Constant)	49.96	0.89		56.21	0.000
Rumination	0.34	0.082	0.153	4.18	0.000
BDI (Beck)	−0.09	0.027	−0.120	−3.28	0.001

In the final model, higher rumination scores were associated with better diet quality (B = 0.341, β = 0.153, *p* < 0.001), while greater depressive symptoms (BDI) were associated with poorer diet quality (B = −0.090, β = −0.120, *p* = 0.001). Given the small, explained variance, these associations should be viewed as tentative signals rather than confirmatory effects.

### Testing the second hypothesis

A stepwise regression analysis was conducted to identify which variables most significantly best predict eating behaviors (AEBQ total score), using the Akaike information criterion for variable selection and verifying that all variance inflation factors remained below 2.0. The initial model included the following predictors: Beck Anxiety Inventory (BAI), Shirom-Melamed Burnout Measure (Shirom Total), the SCL, selected WHOQOL subscales, and specific CERQ subscales (Focus on the Positive, Positive Reappraisal, Positive Refocusing, Acceptance, Catastrophizing, and Social Relationships). The primary aim was to determine how these variables relate to AEBQ.

The exploratory stepwise model showed a moderate overall association with AEBQ (R = 0.404, Adj. R^2^ = 0.155, *F*(8,797) = 19.47, *p* < 0.001), indicating that the selected predictors collectively accounted for 15.5% of variance. The results are hypothesis-generating. The standard error of the estimate (0.357) reflects the average distance by which the observed AEBQ scores deviate from the regression line (see Table 7).

**Table 7:** Model summary for stepwise regression predicting AEBQ.

R	R square	Adjusted R square	Std. error of the estimate
0.404^h^	0.163	0.155	0.357

The ANOVA results (*F*(8, 797) = 19.466, *p* < 0.001) indicate that the overall regression model is statistically significant. Specifically, the regression component accounted for 19.795 of the total 121.103 sum of squares, while the residual variance was 101.308. These findings suggest that, collectively, the included predictors significantly explain variance in the dependent variable, AEBQ (see Table 8).

**Table 8:** ANOVA for stepwise regression predicting AEBQ.

Variable	Sum of squares	df	Mean square	F	Sig.
Regression	19.795	8	2.474	19.466	0.000^i^
Residual	101.308	797	0.127		
Total	121.103	805			

A regression analysis revealed that multiple variables significantly contribute to explaining variation in the dependent variable, AEBQ. Anxiety (BAI) was positively associated with AEBQ (B = 0.006, *β* = 0.182, t = 4.379, *p* < 0.001). Similarly, focus on the positive was positively associated with AEBQ (B = 0.021, β = 0.186, t = 4.359, *p* < 0.001).

In contrast, positive reappraisal was inversely associated with AEBQ (B = −0.017, β = −0.156, t = −3.624, *p* < 0.001), while positive refocusing was positively associated (B = 0.018, β = 0.173, t = 3.864, *p* < 0.001). Acceptance was negatively associated with AEBQ (B = −0.013, β = −0.115, t = −2.574, *p* = 0.010).

Catastrophizing was positively associated with AEBW (B = 0.013, β = 0.107, t = 2.696, *p* = 0.007). Social relationships (B = 0.042, β = 0.095, t = 2.622, *p* = 0.009) and Shirom Total (B = 0.002, β = 0.096, t = 2.318, *p* = 0.021) also showed positive associations with AEBQ (see Table 9).

**Table 9:** Coefficients for predictors of AEBQ.

Variable	Unstandardized coefficients B	Std. error	Standardized coefficients beta	t	Sig.
Constant	2.384	0.095		25.228	0.000
BAI (anxiety)	0.006	0.001	0.182	4.379	0.000
Focus on the positive	0.021	0.005	0.186	4.359	0.000
Positive reappraisal	−0.017	0.005	−0.156	−3.624	0.000
Positive refocusing	0.018	0.005	0.173	3.864	0.000
Acceptance	−0.013	0.005	−0.115	−2.574	0.010
Catastrophizing	0.013	0.005	0.107	2.696	0.007
Social relationships	0.042	0.016	0.095	2.622	0.009
Shirom total	0.002	0.001	0.096	2.318	0.021

Among the retained predictors, higher anxiety (BAI), focus on the positive, positive refocusing, catastrophizing, social relationships, and overall burnout (Shirom total) were associated with more extreme or dysregulated eating behaviors (higher AEBQ scores). In contrast, greater use of positive reappraisal and acceptance corresponded to lower AEBQ scores. These findings underscore the multifaceted ways in which both adaptive and maladaptive coping strategies relate to eating behavior patterns in Czech young adults.

## Discussion

This study is the first census-matched analysis in Central Europe to examine how discrete cognitive emotion-regulation strategies relate to both overall diet quality (DQI-I) and eating behaviors (AEBQ) among young adults. In a representative Czech sample, we observed three key patterns: (1) Rumination was paradoxically associated with higher diet quality, (2) depressive symptoms were associated with lower diet quality, and (3) anxiety, catastrophizing, and “focus on the positive” were linked to stronger food-approach behaviors, whereas positive reappraisal and acceptance were associated with more regulated eating.

Together, these findings highlight specific cognitive-emotional mechanisms that shape dietary behavior during a critical developmental stage, thereby extending current understanding in nutritional psychiatry and illustrating the complex interplay between psychological processes and nutrition in young adulthood.

### Psychological predictors of dietary quality

Contrary to our initial hypothesis, higher levels of rumination were associated with better overall diet quality (*β* = 0.153, *p* < 0.001). At face value, this appears counterintuitive as rumination is typically regarded as a maladaptive strategy to distress, depressive symptomatology, and emotional eating (21). However, several mechanisms may help explain this paradoxical finding.

First, in non-clinical populations, rumination may overlap with perseverative monitoring and planning tendencies, which can foster structure in daily routines. For instance, repetitive thought patterns may reinforce behaviors such as meal planning, careful food selection, or adherence to external dietary norms. This perspective aligns with findings suggesting that repetitive thinking is not uniformly maladaptive and, in some contexts, may support organized, rule-guided behavior (10, 22).

Second, rumination has been linked to self-focused cognitive vigilance. In community studies, it mediated associations between dieting and both uncontrolled and emotional eating, suggesting that rumination contributes not only to maladaptive responses but also to diet-monitoring behaviors (23, 24). Similarly, ecological momentary assessment research indicates that momentary rumination predicts episodes of emotional eating, especially in individuals of normal weight, pointing to rumination’s role in moment-to-moment cognitive vigilance around eating (23). In such contexts, rumination may operate less as a trigger of dysregulation and more as a mechanism maintaining heightened attention to food-related behaviors.

Third, personality research offers another interpretation. Rumination shares variance with traits such as conscientiousness and self-discipline, which are robust predictors of healthier dietary patterns and higher adherence to nutritional guidelines (25). This overlap may help explain which, in our sample, rumination tracked with better diet quality. Put differently, repetitive cognitive engagement with one’s behavior may, for some individuals, function as a self-regulatory tool rather than as a vulnerability factor.

By contrast, depressive symptoms were associated with poorer diet quality (BDI; *β* = −0.120, *p* = 0.001), consistent with cross-cultural evidence that mood disturbance often coincides with less healthy eating (26). However, the directionality of this association remains unclear.

It is important to note, however, that the overall model explained only 2.4% of variance in DQI-I scores (Adj. R^2^ = 0.024, f^2^ = 0.025). While statistically significant, this small effect size suggests that rumination and depressive symptoms, though meaningful predictors, account for only a limited proportion of variability in diet quality. This underscores the complexity of dietary behaviors, which are shaped not only by psychological processes but also by socioeconomic, cultural, and environmental factors.

Taken together, these results highlight the dual nature of rumination. While traditionally viewed as maladaptive, our exploratory findings raise the possibility that, under certain circumstances, rumination may serve a self-regulatory role, supporting consistency and adherence to dietary routines. This interpretation is consistent with a growing literature arguing that the functional meaning of cognitive strategies is context-dependent: The same cognitive process can be either detrimental or adaptive depending on the broader behavioral and environmental framework in which it occurs, although this interpretation remains tentative.

### Psychological predictors of eating behaviors

Consistent with prior literature, depressive symptoms were associated with poorer diet quality, underscoring how low mood disrupts motivation and self-regulation around eating (27, 43). More notably, however, were predictors of eating-behavior patterns. Anxiety showed the strongest positive association with dysregulated eating (BAI; *β* = 0.182, *p* < 0.001), aligning with evidence that stress and anxious arousal can trigger emotional eating and heightened food cue reactivity (28). Maladaptive strategies, such as catastrophizing (β = 0.107, *p* = 0.007), were associated with greater food-approach tendencies, reinforcing prior work on the role of exaggerated negative cognitions in affect-driven eating (29).

In contrast, adaptive strategies were associated with lower food-approach scores. Both positive reappraisal (β = −0.156, *p* < 0.001) and acceptance (β = −0.115, *p* = 0.010) were associated with lower AEBQ food approach scores, consistent with intervention studies showing that reappraisal- and mindfulness-based approaches reduce emotional and binge eating (30). These findings suggest that cultivating adaptive coping skills may buffer against stress-induced overeating (31).

At the same time, our data revealed a nuanced picture. The CERQ strategies “focus on the positive” (β = 0.186, *p* < 0.001) and “positive refocusing” (β = 0.173, *p* < 0.001) were unexpectedly linked to greater food approach behaviors. One possible explanation is that, in high-stress contexts, shifting attention toward positive stimuli may enhance reward sensitivity, thereby increasing responsiveness to palatable foods. Social reinforcement could also play a role as positively valenced coping may be expressed through hedonic or communal eating. This aligns with the findings that reward-seeking pathways can override otherwise adaptive strategies, depending on the situational context (27, 32).

Taken together, our model explained 15.5% of the variance in AEBQ scores (Adj. R^2^ = 0.155), representing a modest but meaningful effect given the multifactorial nature of eating behavior. These exploratory associations point to the potential value of interventions addressing depressive and anxiety symptoms, particularly those using cognitive-behavioral techniques to reduce catastrophizing while fostering acceptance and reappraisal, may simultaneously benefit mood and dietary regulation. Meal-planning skills training could further capitalize on the attentional focus characteristic of perseverative thinking, while mindfulness-based approaches may strengthen adaptive regulation without inadvertently heightening sensitivity to food cues. Indeed, mindfulness meditation has been shown to reduce stress-related overeating and improve interoceptive awareness, a key process in modulating emotional triggers of eating (33). Future trials are needed to test whether such approaches improve both mood and eating regulation.

Finally, these findings must be considered within the broader dietary and psychological transitions experienced by young adults in Central and Eastern Europe (CEE). For instance, Dalecká et al. (44)conducted two large-scale Czech surveys during the COVID-19 pandemic and found that declines in mental health status were closely tied to deteriorations in dietary habits, underscoring that psychological distress can precede unhealthy food behaviors in this region. Moreover, studies of Czech and Slovak students indicate prevalent suboptimal diet quality, low consumption of fruit and vegetables, and high intake of processed foods (7, 8). These trends reflect structural shifts in dietary habits during transitional life stages. Our results suggest that emotion regulation strategies may shape how young adults navigate these environmental pressures, mediating the interplay between changing food environments and psychological vulnerability.

## Strengths, limitations, and future directions

This study has several strengths. It draws on a large, census-matched sample of Czech young adults, enhancing representativeness and external validity. The use of well-validated psychological instruments alongside culturally adapted dietary and eating-behavior measures allowed for a comprehensive assessment of both mental health symptoms and cognitive emotion-regulation processes. By integrating multiple domains, the study provides robust insights into the psychological mechanisms underpinning dietary quality and eating behavior, with potential applicability to similar young-adult populations in Central and Eastern Europe.

From an applied perspective, the findings suggest opportunities for integrated interventions that combine nutritional education with emotion-regulation training. University health programs, for example, might benefit from modules that:

- Target catastrophizing and anxiety-driven eating, which were associated with dysregulated food-approach tendencies, and
- Promote positive reappraisal and acceptance/mindfulness techniques, both of which have demonstrated efficacy in reducing emotional and binge eating (30, 31).

Such integrated approaches may enhance the effectiveness of nutrition and mental health promotion efforts among young adults, a population navigating critical lifestyle transitions.

Several limitations must be acknowledged:

1. The Czech adaptation of the FFQ and AEBQ, while showing acceptable pilot reliability, has not undergone full psychometric validation, which should be addressed in subsequent studies.
2. Reliance self-reported dietary data and computation of DQI-I from FFQ responses raise concerns about recall and social desirability bias; future studies leveraging biomarkers or dietary recalls could help validate the findings.
3. The cross-sectional design limits causal inference. Longitudinal and experimental studies, including randomized controlled trials testing emotion-regulation interventions, are needed to clarify temporal and causal pathways.
4. The use of stepwise regression approach was exploratory (though predictors showed low multicollinearity), so the results should be interpreted as hypothesis-generating rather than confirmatory.

In summary, this study highlights the nuanced interplay between mental health and nutritional behaviors in young adulthood. It underscores the potential value of holistic interventions that simultaneously address cognitive-emotional regulation and dietary habits, thereby maximizing benefits for psychological wellbeing and physical health.

## Conclusion

Our findings highlight multifaceted links between mental health and nutrition, indicating that both maladaptive (e.g., rumination, catastrophizing, and depressive symptoms) and adaptive (e.g., positive reappraisal and acceptance) cognitive–emotional factors were associated with diet quality and eating behaviors in Czech young adults. These associations should be regarded as exploratory and hypothesis-generating. Although effect sizes were modest, the identified relationships suggest that combining mental-health interventions—such as cognitive-behavioral strategies targeting rumination and depression—with practical nutritional guidance may yield greater benefits than addressing either domain alone. Future research should employ longitudinal designs and fully validate Czech adaptations of key instruments to clarify causal pathways and optimize targeted, integrative programs that enhance both psychological resilience and dietary wellbeing.

By embedding cognitive-emotional factors within the specific dietary environment of Czech young adults, our findings reveal nuanced pathways linking emotion regulation to diet quality and eating behavior. Rumination, when channeled toward health-conscious cognition, may in some context be linked to better dietary outcomes, although this remains tentative. Targeting maladaptive cognitive strategies while bolstering adaptive ones like reappraisal and acceptance offers promising leverage points for nutritional psychiatry and health promotion in transitional young adult populations.

## Data Availability

The datasets presented in this article are not readily available because consent to share the data with third parties was not obtained from participants.
