# Supplementary material for: Exploring the relationship between dietary quality, eating behavior, and mental health among young adults
Source: Front Nutr. 2025 Nov 3;12:1598260. doi: 10.3389/fnut.2025.1598260 (PMC12621320; doi:10.3389/fnut.2025.1598260)
Supplement: Supplementary file 1 [file Table_1.docx]

**Annex**

**Table S1**. Pearson Correlations Among DQI, AEBQ, WHOQOL, CERQ, and Mental Health Scales

| **Correlations** | | **n=1030** |  |  |  |  |  | **WHOQOL** | | | | **CERQ** | | | | | | | | |
| --- | --- | --- | --- | --- | --- | --- | --- | --- | --- | --- | --- | --- | --- | --- | --- | --- | --- | --- | --- | --- |
|  |  | DQI | AEBQ Total | Shirom Total | BDI | BAI | SCL | Physical healht | Mental health | Scoal relat. | Environment | Self-Blame | Acceptance | Rumination | Blame | Positive Reappraisal | Planning | Positive Refocusing | Focus on the Positive | Catastrophizing |
| DQI | Pearson Correlation | 1 | -,086^**^ | -0.038 | -,084^**^ | -0.021 | -0.047 | ,068^*^ | ,091^**^ | ,062^*^ | ,111^**^ | 0.003 | ,076^*^ | ,114^**^ | ,088^**^ | ,130^**^ | ,134^**^ | ,104^**^ | -0.034 | 0.004 |
|  | Sig. (2-tailed) |  | 0.006 | 0.276 | 0.007 | 0.497 | 0.132 | 0.029 | 0.004 | 0.046 | 0.000 | 0.912 | 0.015 | 0.000 | 0.005 | 0.000 | 0.000 | 0.001 | 0.281 | 0.893 |
| AEBQ Total | Pearson Correlation | -,086^**^ | 1 | ,243^**^ | ,237^**^ | ,263^**^ | ,266^**^ | -,080^*^ | -,117^**^ | -0.050 | -,173^**^ | ,117^**^ | 0.026 | ,123^**^ | 0.040 | -0.037 | -0.009 | 0.057 | ,255^**^ | ,185^**^ |
|  | Sig. (2-tailed) | 0.006 |  | 0.000 | 0.000 | 0.000 | 0.000 | 0.010 | 0.000 | 0.108 | 0.000 | 0.000 | 0.414 | 0.000 | 0.195 | 0.238 | 0.763 | 0.068 | 0.000 | 0.000 |
| Shirom Total | Pearson Correlation | -0.038 | ,243^**^ | 1 | ,684^**^ | ,574^**^ | ,642^**^ | -,472^**^ | -,490^**^ | -,389^**^ | -,455^**^ | ,343^**^ | ,109^**^ | ,260^**^ | -,092^**^ | -0.059 | -,079^*^ | -0.002 | ,349^**^ | ,171^**^ |
|  | Sig. (2-tailed) | 0.276 | 0.000 |  | 0.000 | 0.000 | 0.000 | 0.000 | 0.000 | 0.000 | 0.000 | 0.000 | 0.002 | 0.000 | 0.009 | 0.097 | 0.025 | 0.947 | 0.000 | 0.000 |
| BDI (Beck) | Pearson Correlation | -,084^**^ | ,237^**^ | ,684^**^ | 1 | ,700^**^ | ,799^**^ | -,442^**^ | -,548^**^ | -,468^**^ | -,481^**^ | ,397^**^ | ,130^**^ | ,291^**^ | -,111^**^ | -,071^*^ | -,132^**^ | -0.045 | ,476^**^ | ,214^**^ |
|  | Sig. (2-tailed) | 0.007 | 0.000 | 0.000 |  | 0.000 | 0.000 | 0.000 | 0.000 | 0.000 | 0.000 | 0.000 | 0.000 | 0.000 | 0.000 | 0.022 | 0.000 | 0.149 | 0.000 | 0.000 |
| BAI (úzkost) | Pearson Correlation | -0.021 | ,263^**^ | ,574^**^ | ,700^**^ | 1 | ,846^**^ | -,287^**^ | -,392^**^ | -,343^**^ | -,456^**^ | ,341^**^ | ,142^**^ | ,289^**^ | -0.019 | 0.026 | 0.002 | 0.037 | ,417^**^ | ,245^**^ |
|  | Sig. (2-tailed) | 0.497 | 0.000 | 0.000 | 0.000 |  | 0.000 | 0.000 | 0.000 | 0.000 | 0.000 | 0.000 | 0.000 | 0.000 | 0.539 | 0.413 | 0.960 | 0.238 | 0.000 | 0.000 |
| SCL | Pearson Correlation | -0.047 | ,266^**^ | ,642^**^ | ,799^**^ | ,846^**^ | 1 | -,343^**^ | -,476^**^ | -,444^**^ | -,483^**^ | ,419^**^ | ,173^**^ | ,363^**^ | -0.031 | 0.016 | -0.025 | 0.041 | ,508^**^ | ,311^**^ |
|  | Sig. (2-tailed) | 0.132 | 0.000 | 0.000 | 0.000 | 0.000 |  | 0.000 | 0.000 | 0.000 | 0.000 | 0.000 | 0.000 | 0.000 | 0.324 | 0.602 | 0.431 | 0.190 | 0.000 | 0.000 |
| Physical Health | Pearson Correlation | ,068^*^ | -,080^*^ | -,472^**^ | -,442^**^ | -,287^**^ | -,343^**^ | 1 | ,541^**^ | ,423^**^ | ,487^**^ | -,135^**^ | -0.017 | -,085^**^ | ,188^**^ | ,164^**^ | ,153^**^ | ,062^*^ | -,162^**^ | -0.009 |
|  | Sig. (2-tailed) | 0.029 | 0.010 | 0.000 | 0.000 | 0.000 | 0.000 |  | 0.000 | 0.000 | 0.000 | 0.000 | 0.587 | 0.007 | 0.000 | 0.000 | 0.000 | 0.047 | 0.000 | 0.776 |
| Mental Health | Pearson Correlation | ,091^**^ | -,117^**^ | -,490^**^ | -,548^**^ | -,392^**^ | -,476^**^ | ,541^**^ | 1 | ,543^**^ | ,620^**^ | -,225^**^ | 0.010 | -,093^**^ | ,274^**^ | ,238^**^ | ,270^**^ | ,154^**^ | -,285^**^ | -0.045 |
|  | Sig. (2-tailed) | 0.004 | 0.000 | 0.000 | 0.000 | 0.000 | 0.000 | 0.000 |  | 0.000 | 0.000 | 0.000 | 0.742 | 0.003 | 0.000 | 0.000 | 0.000 | 0.000 | 0.000 | 0.149 |
| Social Relat. | Pearson Correlation | ,062^*^ | -0.050 | -,389^**^ | -,468^**^ | -,343^**^ | -,444^**^ | ,423^**^ | ,543^**^ | 1 | ,502^**^ | -,192^**^ | -0.014 | -,130^**^ | ,208^**^ | ,150^**^ | ,143^**^ | ,132^**^ | -,268^**^ | -,110^**^ |
|  | Sig. (2-tailed) | 0.046 | 0.108 | 0.000 | 0.000 | 0.000 | 0.000 | 0.000 | 0.000 |  | 0.000 | 0.000 | 0.647 | 0.000 | 0.000 | 0.000 | 0.000 | 0.000 | 0.000 | 0.000 |
| Environment | Pearson Correlation | ,111^**^ | -,173^**^ | -,455^**^ | -,481^**^ | -,456^**^ | -,483^**^ | ,487^**^ | ,620^**^ | ,502^**^ | 1 | -,100^**^ | ,103^**^ | -0.025 | ,168^**^ | ,185^**^ | ,211^**^ | ,185^**^ | -,283^**^ | -,170^**^ |
|  | Sig. (2-tailed) | 0.000 | 0.000 | 0.000 | 0.000 | 0.000 | 0.000 | 0.000 | 0.000 | 0.000 |  | 0.001 | 0.001 | 0.431 | 0.000 | 0.000 | 0.000 | 0.000 | 0.000 | 0.000 |
| Self-Blame | Pearson Correlation | 0.003 | ,117^**^ | ,343^**^ | ,397^**^ | ,341^**^ | ,419^**^ | -,135^**^ | -,225^**^ | -,192^**^ | -,100^**^ | 1 | ,447^**^ | ,608^**^ | ,106^**^ | ,346^**^ | ,262^**^ | ,302^**^ | ,515^**^ | ,230^**^ |
|  | Sig. (2-tailed) | 0.912 | 0.000 | 0.000 | 0.000 | 0.000 | 0.000 | 0.000 | 0.000 | 0.000 | 0.001 |  | 0.000 | 0.000 | 0.001 | 0.000 | 0.000 | 0.000 | 0.000 | 0.000 |
| Acceptance | Pearson Correlation | ,076^*^ | 0.026 | ,109^**^ | ,130^**^ | ,142^**^ | ,173^**^ | -0.017 | 0.010 | -0.014 | ,103^**^ | ,447^**^ | 1 | ,521^**^ | ,396^**^ | ,552^**^ | ,589^**^ | ,603^**^ | ,317^**^ | ,255^**^ |
|  | Sig. (2-tailed) | 0.015 | 0.414 | 0.002 | 0.000 | 0.000 | 0.000 | 0.587 | 0.742 | 0.647 | 0.001 | 0.000 |  | 0.000 | 0.000 | 0.000 | 0.000 | 0.000 | 0.000 | 0.000 |
| Rumination | Pearson Correlation | ,114^**^ | ,123^**^ | ,260^**^ | ,291^**^ | ,289^**^ | ,363^**^ | -,085^**^ | -,093^**^ | -,130^**^ | -0.025 | ,608^**^ | ,521^**^ | 1 | ,235^**^ | ,495^**^ | ,402^**^ | ,385^**^ | ,525^**^ | ,379^**^ |
|  | Sig. (2-tailed) | 0.000 | 0.000 | 0.000 | 0.000 | 0.000 | 0.000 | 0.007 | 0.003 | 0.000 | 0.431 | 0.000 | 0.000 |  | 0.000 | 0.000 | 0.000 | 0.000 | 0.000 | 0.000 |
| Blame | Pearson Correlation | ,088^**^ | 0.040 | -,092^**^ | -,111^**^ | -0.019 | -0.031 | ,188^**^ | ,274^**^ | ,208^**^ | ,168^**^ | ,106^**^ | ,396^**^ | ,235^**^ | 1 | ,606^**^ | ,566^**^ | ,522^**^ | ,161^**^ | ,307^**^ |
|  | Sig. (2-tailed) | 0.005 | 0.195 | 0.009 | 0.000 | 0.539 | 0.324 | 0.000 | 0.000 | 0.000 | 0.000 | 0.001 | 0.000 | 0.000 |  | 0.000 | 0.000 | 0.000 | 0.000 | 0.000 |
| Positive Reappraisal | Pearson Correlation | ,130^**^ | -0.037 | -0.059 | -,071^*^ | 0.026 | 0.016 | ,164^**^ | ,238^**^ | ,150^**^ | ,185^**^ | ,346^**^ | ,552^**^ | ,495^**^ | ,606^**^ | 1 | ,729^**^ | ,598^**^ | ,193^**^ | ,274^**^ |
|  | Sig. (2-tailed) | 0.000 | 0.238 | 0.097 | 0.022 | 0.413 | 0.602 | 0.000 | 0.000 | 0.000 | 0.000 | 0.000 | 0.000 | 0.000 | 0.000 |  | 0.000 | 0.000 | 0.000 | 0.000 |
| Planning | Pearson Correlation | ,134^**^ | -0.009 | -,079^*^ | -,132^**^ | 0.002 | -0.025 | ,153^**^ | ,270^**^ | ,143^**^ | ,211^**^ | ,262^**^ | ,589^**^ | ,402^**^ | ,566^**^ | ,729^**^ | 1 | ,666^**^ | ,108^**^ | ,208^**^ |
|  | Sig. (2-tailed) | 0.000 | 0.763 | 0.025 | 0.000 | 0.960 | 0.431 | 0.000 | 0.000 | 0.000 | 0.000 | 0.000 | 0.000 | 0.000 | 0.000 | 0.000 |  | 0.000 | 0.001 | 0.000 |
| Positive Refocusing | Pearson Correlation | ,104^**^ | 0.057 | -0.002 | -0.045 | 0.037 | 0.041 | ,062^*^ | ,154^**^ | ,132^**^ | ,185^**^ | ,302^**^ | ,603^**^ | ,385^**^ | ,522^**^ | ,598^**^ | ,666^**^ | 1 | ,109^**^ | ,203^**^ |
|  | Sig. (2-tailed) | 0.001 | 0.068 | 0.947 | 0.149 | 0.238 | 0.190 | 0.047 | 0.000 | 0.000 | 0.000 | 0.000 | 0.000 | 0.000 | 0.000 | 0.000 | 0.000 |  | 0.000 | 0.000 |
| Focus on the Positive | Pearson Correlation | -0.034 | ,255^**^ | ,349^**^ | ,476^**^ | ,417^**^ | ,508^**^ | -,162^**^ | -,285^**^ | -,268^**^ | -,283^**^ | ,515^**^ | ,317^**^ | ,525^**^ | ,161^**^ | ,193^**^ | ,108^**^ | ,109^**^ | 1 | ,523^**^ |
|  | Sig. (2-tailed) | 0.281 | 0.000 | 0.000 | 0.000 | 0.000 | 0.000 | 0.000 | 0.000 | 0.000 | 0.000 | 0.000 | 0.000 | 0.000 | 0.000 | 0.000 | 0.001 | 0.000 |  | 0.000 |
| Catastrophizing | Pearson Correlation | 0.004 | ,185^**^ | ,171^**^ | ,214^**^ | ,245^**^ | ,311^**^ | -0.009 | -0.045 | -,110^**^ | -,170^**^ | ,230^**^ | ,255^**^ | ,379^**^ | ,307^**^ | ,274^**^ | ,208^**^ | ,203^**^ | ,523^**^ | 1 |
|  | Sig. (2-tailed) | 0.893 | 0.000 | 0.000 | 0.000 | 0.000 | 0.000 | 0.776 | 0.149 | 0.000 | 0.000 | 0.000 | 0.000 | 0.000 | 0.000 | 0.000 | 0.000 | 0.000 | 0.000 |  |
